# The development of VISUALISE: a co-designed, theory-based self-management intervention for stroke-related visual impairment

**DOI:** 10.1080/21642850.2026.2753947

**Published:** 2026-11-07

**Authors:** Ciara Ryan, Katie Elliott, Maggie Lawrence, Lisa Kidd, Christine Hazelton

**Affiliations:** a School of Health and Social Care, Edinburgh Napier University, UK; b School of Health and Life Sciences, Glasgow Caledonian University, UK; c Department of Nursing, Community and Public Health, Glasgow Caledonian University, UK

**Keywords:** Stroke, visual impairment, self-management, intervention mapping, co-design

## Abstract

**Background:**

Stroke‑related visual impairment (SRVI) affects up to 65% of individuals post-stroke and has sustained consequences for confidence, participation, and emotional well-being. While self‑management is increasingly emphasised in stroke policy, existing programmes do not specifically address the long‑term needs associated with stroke-related visual impairment. This study aimed to develop VISUALISE, a theory‑driven, evidence‑informed, and co‑designed self‑management intervention tailored for stroke-related visual impairment.

**Methods:**

Intervention Mapping, underpinned by Social Cognitive Theory, guided intervention design and drew on four evidence sources: (1) a mixed‑method systematic review; (2) a qualitative study of lived experience and unmet needs; (3) five stakeholder focus groups involving people with lived experience, professionals, caregivers, and academics; and (4) Behaviour Change Technique mapping using the Theory and Techniques Tool. Data were analysed using a framework approach aligned with Intervention Mapping stages.

**Results:**

VISUALISE comprises nine weekly peer‑led sessions supported by an accessible workbook. The intervention targets four domains: information and education, practical support, peer and emotional support, and strengthening confidence. Key behaviour change techniques include modelling, structured goal‑setting, and self‑monitoring. Co‑design contributions shaped specific features such as a readiness assessment, protected pre‑session peer interaction, balanced peer narratives, tailored accessibility adaptations, and optional booster sessions. A TIDieR specification outlines materials, procedures, tailoring, delivery, and facilitator training.

**Conclusion:**

VISUALISE is a novel, theory‑driven, co‑designed self‑management intervention tailored for stroke‑related visual impairment. The intervention specifies hypothesised mechanisms of action and practical implementation considerations. Future research should evaluate acceptability, fidelity, and effectiveness in real‑world settings.

## Introduction

Stroke-related visual impairment (SRVI), including eye movement disorders, visual field loss, and visual perceptual difficulties, affects up to 65% of people following a stroke (Hepworth et al., 2021). Evidence indicated that although some degree of spontaneous recovery may occur during the early post‑stroke period, many individuals experience long‑term or permanent impairment, with highly variable recovery trajectories and uncertain prognosis (Ali et al., 2013). SRVI is associated with increased anxiety and depression, reduced health‑related quality of life, impaired mobility and reading, and diminished independence (Hepworth & Rowe, 2016; Sand et al., 2016). Qualitative studies highlight fear of falling, loss of confidence, identity disruption, and unmet informational and emotional needs (Falkenberg et al., 2020; Hazelton et al., 2019; Rowe, 2017).

Despite its prevalence and impact, SRVI remains inconsistently assessed and managed. Within Scotland, stroke rehabilitation is typically delivered across acute, community, and longer-term care settings in line with the National Clinical Guideline for Stroke for the UK and Ireland (2023). Scottish Government (2023) further emphasises the importance of person-centred, holistic rehabilitation delivered across health, social care, and third-sector services, with a focus on supported self-management, goal-setting, and opportunities to re-access rehabilitation over time. National and international guidelines, including the recent European Stroke Organisation (ESO) Guideline (Rowe et al., 2025), recommend systematic vision screening within one week of stroke onset and appropriate management of post-stroke visual impairment. However, evidence indicates that access to specialist visual rehabilitation and longer-term support remains variable, and individuals may need to navigate a fragmented mix of statutory and third-sector services during recovery (Rowe et al., 2013; Ryan et al., 2026). Many individuals receive no formal assessment, limited or inaccurate information, and no referral to specialist services (Rowe et al., 2013). Existing rehabilitation often focuses onSingh short-term restorative or compensatory strategies, with comparratively limited support for long-term adaptation, emotional adjustment, or self-management (Hanna et al., 2017).

Self‑management is increasingly positioned as a core component of high‑quality stroke care (Lynch et al., 2025), and involves supporting individuals to build the skills, knowledge, and confidence required to live well with a long-term condition (de longh et al., 2015; De Silva, 2011). Evidence from general stroke populations shows improvements in activities of daily living, functional mobility, and self‑efficacy, although quality‑of‑life gains remain inconsistent (Fryer et al., 2016; Lynch et al., 2025). Studies in non‑stroke visual‑impairment populations similarly report modest improvements in wellbeing and self‑efficacy (Malkin et al., 2025). However, existing interventions are typically multicomponent, theoretically inconsistent, and poorly specified, making active mechanisms difficult to identify (Boger et al., 2013; Fryer et al., 2016; Malkin et al., 2025). To our knowledge, no published self‑management intervention has been specifically designed to address the psychological and functional challenges unique to stroke-related visual impairment.

These gaps highlight the need for a structured, theory‑driven, co‑designed intervention tailored for stroke-related visual impairment. Intervention Mapping (Bartholomew et al., 1998) was chosen because it integrates behavioural theory, empirical evidence, and stakeholder perspectives across iterative planning stages. Accordingly, this study aimed to: (1) identify the core components of an SRVI‑specific self‑management intervention grounded in lived experience; (2) determine stakeholder‑informed preferences for content, materials, delivery, and accessibility; and (3) produce a comprehensive, reproducible intervention specification using the Template for Intervention Description and Replication (TIDieR) checklist (Hoffmann et al., 2014) to support future implementation and evaluation.

## Methods

### Design

This study was conducted within the development phase of the Medical Research Council (MRC) Framework for Complex Interventions (Craig & Petticrew, 2013; Skivington et al., 2021), which emphasises the importance of theory-driven development, iterative refinement, and transparency in decision-making. Intervention Mapping (Bartholomew et al., 1998) was informed by two earlier studies, a mixed-method systematic review (Ryan, 2023) and a qualitative needs study (Ryan et al., 2026). Findings from these earlier studies informed the needs assessment, whereas the coding, mapping, logic models, matrices of change objectives, and TIDieR specification reported here are original outputs of the present study.

This paper reports Intervention Mapping Stages 1–4 (needs assessment, logic model of change, method selection, and programme design). Stages 5–6 (implementation and evaluation planning) were beyond the scope of this development phase and will be addressed in future work. An overview of evidence inputs and outputs is provided in Table 1. Detailed Intervention Mapping outputs, including the integrated logic model, applications of Social Cognitive Theory, and the full TIDieR intervention specification, are provided in the Supplementary Material (Supplementary Tables S1–S3).

### Ethics statement

Ethical approval was granted by the Nursing and Community Health Departmental Ethics Committee at Glasgow Caledonian University (Ref: HLS/NCH/20/048). Fully informed written consent was gained from all participants who took part in the study.

**Table 1.** Integrated overview of Intervention Mapping stages, evidence inputs, focus groups, and outputs.

Intervention Mapping Stage	Evidence input(s)	Focus group	Primary purpose of focus groups	Output(s)
Stage 1: Needs assessment & Logic Model of the Problem	Mixed‑method systematic review; qualitative study identifying and prioritising unmet needs among people with stroke-related visual impairment	FG1	Identify priority unmet needs and associated personal and environmental determinants.	Logic Model of the Problem (see Table 3).
Stage 2: Outcomes, Performance & Change Objectives (Logic Model of Change)	Logic Model of the Problem (see Table 3).	FG2	Specify behavioural and environmental outcomes; define performance objectives and determinants; link these to SCT mechanisms.	Logic Model of the Problem and Change Objectives (see Table 3).
Stage 3: Theory-based Methods & Practical Applications	Theory & Techniques Tool mapping SCT constructs to BCTs; stakeholder feedback	FG3	Select empirically supported BCTs and translate them into feasible SRVI‑specific applications.	SCT–BCT–application matrix linking mechanisms of action, behaviour change techniques, and intervention components (see Table 4)
Stage 4: Programme design (TIDieR specification)	Stage 3 method selections; reporting structured by TIDieR; delivery, dose, and accessibility preferences from earlier stages.	FG4–FG5	Finalise programme structure, delivery mode, dose, materials, tailoring, and provider roles; confirm feasibility considerations.	VISUALISE intervention specification using the TIDier template (Hoffmann et al., 2014) (see Table 5)
Stage 5: Adoption & Implementation planning	Not undertaken in this study	N/A	Beyond the scope of this development phase.	To be addressed in future implementation research.
Stage 6: Evaluation planning	Not undertaken in this study	N/A		

Abbreviations: *SCT = Social Cognitive Theory; BCT = behaviour change technique; TIDieR = Template for Intervention Description and Replication; SRVI = stroke-related visual impairment.*

### Stakeholder recruitment

Stakeholders were purposively selected to ensure representation across lived-experience, clinical, psychological, and implementation perspectives. A Patient and Public Involvement (PPI) advisory group, including three individuals with lived experience of SRVI (5–20 years post‑stroke), participated throughout all stages of intervention development, including each phase of the Intervention Mapping process. Professionals and caregivers were recruited via clinical networks, organisations, and existing research contacts.

Eligibility criteria for professionals included a minimum of five years' professional experience and current involvement in stroke rehabilitation, vision rehabilitation, or psychosocial support services. These criteria were intended to ensure that contributors possessed relevant expertise in service delivery, behavioural change, and psychosocial aspects of care.

Stakeholders were based predominantly in Scotland, with the exception of one lived-experience contributor based in England. As such, stakeholder perspectives were grounded primarily in the Scottish health and social care context, while also reflecting wider UK rehabilitation and support pathways. International stakeholders were not actively recruited, as the intervention was being developed for implementation and subsequent feasibility testing within the UK context.

### Data collection

Data were collected through five sequential focus groups, each aligned with the Intervention Mapping stages (see Table 1). All sessions were conducted online due to COVID‑19 restrictions and lasted approximately two hours. The same stakeholder panel was invited to participate across all five sessions, enabling iterative intervention development through repeated engagement over time. Attendance varied across sessions due to availability and is reported in Table 2.

To support engagement and equitable participation, participants received preparatory materials one week before each session, including aims, prompts, summaries of findings, and accessible explanations of key terms. The lead researcher facilitated sessions with two co‑facilitators.

Ground rules emphasised the equal value of lived‑experience and professional perspectives. Given the numerical imbalance between professional and lived experience contributors (see Table 2), several strategies was implemented across all sessions to minimise the risk of power imbalances. Facilitators (CR, KE, CH) actively monitored participation patterns, invited quieter contributors, and explicitly sought and foregroundedlived-experience perspectives during decision-making discussions.

### Data analysis

Data were analysed using a theory‑driven Framework Approach (Gale et al., 2013), supported by NVivo12, and structured to align with each stage of Intervention Mapping. An a priori coding framework combined TIDieR elements (materials, procedures, delivery, tailoring) with core Social Cognitive Theory constructs to support systematic and consistent identification of theoretical mechanisms consistently across all data.

Audio recordings were transcribed verbatim and coded within this framework. Three researchers (CR, KE, CH) independently analysed early transcripts and met regularly to compare interpretations discuss coding decisions, and resolve discrepancies. Coded data were synthesised into outputs for each Intervention Mapping stage, including a needs-based logic model (Stage 1), a logic model of change and matrices of change objectives (Stage 2), a Social Cognitive Theory-Behaviour Change Technique mapping (Stage 3), and a TIDieR structured programme specification (Stage 4). Stakeholders reviewed analytic summaries after each stage to check accuracy, feasibility, and alignment with lived‑experience priorities, enabling iterative refinement across all components.

## Results

Results are organised by Intervention Mapping stage, reporting the analytic outputs and resulting design decisions for Stages 1 to 4.

### Stakeholders

Twelve stakeholders were recruited (see Table 2). All stakeholders were invited to contribute to each stage of intervention development, either through participation in focus groups or by providing feedback via email where attendance was not possible. Two contributors, a stroke rehabilitation nurse and a caregiver, were unable to attend the focus groups due to scheduling constraints and therefore contributed exclusively via email throughout the study. Additional feedback was sought via email from stakeholders who were unable to attend individual focus groups. Overall, the group represented live-experience, clinical, psychological, and methodological perspectives essential to the co-design process.

**Table 2.** Stakeholders and areas of expertise.

Stakeholder group	Expertise	Years of experience/time since stroke	Focus group attendance				
			FG1	FG2	FG3	FG4	FG5
Individuals with lived experience of stroke-related visual impairment (*n* = 3)	Lived experience of adapting to post-stroke visual impairment; prior involvement as advisory contributors. Stroke-related visual impairment types represented include homonymous hemianopia, light sensitivity, and reduced peripheral vision.	5, 12, 20 years post-stroke	*N* = 3	*N* = 3	*N* = 2	*N* = 3	*N* = 3
Eye clinic liaison officer (*n* = 1)	Practical and emotional support for people with visual impairment and their families, with a focus on promoting independence.	5 years	✓	✓	X	✓	✓
Stroke rehabilitation nurse (*n* = 1)	Post discharge stroke care and coordination with statutory and voluntary services.	17 years	E	E	E	E	E
Informal caregiver (*n* = 1)	Lived experience supporting a family member with stroke-related visual impairment in daily activities and rehabilitation.	3 years	E	E	E	E	E
Consultant occupational therapist (*n* = 1)	Clinical expertise in stroke rehabilitation, functional independence, and collaborative goal-setting.	33 years	✓	✓	✓	✓	✓
Low vision rehabilitation officer (*n* = 1)	Delivery and evaluation of rehabilitation interventions for people with stroke-related visual impairment.	20 years	✓	✓	✓	✓	✓
Clinical psychologist (*n* = 1)	Psychological assessment and intervention for individuals with neurological conditions.	20 years	X	E	✓	✓	E
Health psychologist (*n* = 1)	Expertise in psychological aspects of health, including self-management and behaviour change.	11 years	E	✓	X*	X	E
Academic researchers (*n* = 2)	Research experience in stroke rehabilitation, self-management, and implementation science.	7–30 years	*N* = 1	E	*N* = 1	E	E

Note: ✓ = attended focus group; E = contributed feedback via email; X = could not attend focus group/no contribution for that stage. *Following FG3, an additional one-to-one consultation consultation was conducted with the health psychologist following this focus group to provide expert input on the application of Social Cognitive Theory and behaviour change technique mapping.

### Intervention development process

#### Stage 1: Needs assessment and problem analysis

The needs assessment integrated findings from two prior studies conducted as part of the same research programme. First, the mixed-method systematic review (Ryan, 2023) synthesised evidence from 61 studies of self-management interventions for stroke and for visual impairment. Across included interventions, the most frequently reported self-management components were education, peer support, goal-setting, and problem-solving. Among interventions reporting a theoretical basis, Social Cognitive Theory was the only theory identified across both stroke and visual impairment self-management interventions.. However, the review identified substantial heterogeneity in intervention content, dose, and delivery, and several interventions lacked explicit theoretical specification, adequate reporting, and meaningful stakeholder input.

Second, a qualitative interview study with 16 community-dwelling individuals living with SRVI in Scotland (Ryan et al., 2026) used semi-structured interviews and framework analysis to explore lived experience and self-management strategies. Three interconnected themes emerged: multidimensional impacts across practical, emotional, and social domains; self-management strategies developed largely through trial and error; and unmet needs spanning assessment and diagnosis, information provision, practical support, service provision, and peer and emotional support. Participants represented a range of stroke-related visual impairments, including visual field loss, ocular motor disorders, photophobia, visual perceptual difficulties, and related visual symptoms. Despite differences in clinical presentation, common challenges were identified across impairment types, particularly in relation to understanding SRVI, adapting to everyday life, accessing support, maintaining confidence, navigating services, and managing the longer-term consequences of SRVI.

Whilst assessment and diagnosis emerged as the highest priority unmet need, this domain was considered outside the scope of a self‑management intervention, which focuses on building skills, knowledge, and confidence rather than altering diagnostic pathways. Accordingly, Stage 1 carried forward three domains, namely information and education; practical support; and peer and emotional support, as the direct targets for the intervention. A fourth cross‑cutting domain, confidence, was added to reflect its central role in adaptation, engagement and psychological adjustment. Consequently, intervention development focused on addressing these shared unmet needs rather than impairment-specific rehabilitation requirements.

Each domain was underpinned by interacting personal and environmental determinants. For example, limited personal understanding of stroke-related visual impairment interacted with inaccessible materials and fragmented post-discharge referral pathways. A summary logic model is presented in Table 3.Supplementary Table S1 sets out the full set of determinants identified and how each was translated into proposed intervention solutions.

#### Stage 2: Identifying outcomes and change objectives

Drawing on the Stage 1 logic model, stakeholders identified four intended outcomes: (1) improved understanding of stroke‑related visual impairment and available services; (2) increased confidence in using adaptive strategies and assistive devices; (3) development of sustainable peer support networks; and (4) strengthened self‑management and goal‑setting skills for long‑term adaptation. These outcomes were linked to specific behavioural and environmental performance objectives. This formed the basis for identifying change objectives: the specific changes required in knowledge, skills, self-efficacy, expectations, and environmental support needed to achieve each outcome. Table 3 presents the integrated logic model describing domain‑specific determinants and required changes.

**Table 3.** Logic model of the problem and change objectives.

Priority Domain	Key Personal Determinants	Key Environmental Determinants	Core Change Objectives
Information Provision and Education	• Limited understanding of SRVI (nature, causes, functional consequences) • Difficulty articulating SRVI-related challenges to others • Unawareness of available support options and how to access them • Low self-efficacy to seek help or request reasonable accommodations	• Generic, inaccessible, or passively delivered information • Poor timing of information provision (e.g. acute hospital discharge before difficulties are fully apparent) • Limited awareness of SRVI among clinicians and the public • Gap in care between hospital discharge and community service access • Regional inequity in NHS and third-sector provision • Key resources not routinely signposted (e.g. free travel pass, Driver and Vehicle Licensing Agency (DVLA) processes, Citizens Advice)	• Develop a comprehensive understanding of SRVI • Identify and access relevant NHS, third-sector, and peer support services • Confidently explain SRVI to others and request appropriate help or accommodations
Practical Support	• Low self-efficacy to trial or adopt assistive aids and adaptive strategies • Fear of stigma or perceived vulnerability linked to visible aids • Identity disruption related to aid use • Concerns about others’ reactions when using aids in public • Assumptions about IT/digital literacy acting as barriers • Stroke-related co-impairments limiting device use • Variation in pre-stroke technology experience affecting adjustment	• Insufficient therapy time to practise with aids before/after discharge • Lack of demonstration or supported trial opportunities • Financial and resource barriers to acquiring aids and devices	• Identify and trial relevant assistive technologies and adaptive strategies • Access funding sources for aids and devices • Use assistive aids confidently in everyday life
Peer and Emotional Support	• Strong desire for peer connection with others who share lived experience • Pre-existing reluctance or anxiety about peer engagement due to fear of unfavourable social comparison • Misunderstanding of SRVI progression and long-term prognosis • Emotional distress associated with driving licence loss	• Lack of clarity around DVLA notification and assessment processes, causing prolonged uncertainty • Time-limited peer groups with no sustained contact or follow-up • Insufficient professional reassurance delaying psychological adjustment	• Access sustained peer support from others with lived experience of SRVI • Understand DVLA notification and assessment procedures (where relevant) • Feel emotionally supported during adjustment to SRVI
Confidence (cross-cutting domain)	• Low self-efficacy to seek help, self-advocate, or use assistive aids • Reduced sense of identity and self-worth after stroke • Difficulty recognising and valuing retained abilities and personal strengths • Limited opportunity or encouragement to focus on strengths rather than losses	• Limited understanding of SRVI among family members and social networks • Absence of structured, ongoing opportunities to reflect on and celebrate progress	• Recognise and articulate personal strengths and retained abilities • Use structured reflection to monitor and acknowledge progress over time

#### Stage 3: Theory-based methods and practical applications

Social Cognitive Theory constructs were mapped to Behaviour Change Techniques (BCTs) using the Theory and Techniques Tool (Carey et al., 2019). Stakeholders reviewed proposed BCTs for relevance to SRVI and for feasibility within peer-led group delivery (see Table 4). A more detailed mapping of Social Cognitive Theory constructs, sub-constructs, practical applications, and supporting stakeholder quotations is provided in Supplementary Table S2. This ensured that selected methods were theoretically coherent, practically deliverable, and aligned with lived-experience priorities.

**Table 4.** Overview of Social Cognitive Theory, Theory & Techniques Tool behaviour change techniques, and VISUALISE practical applications

Social Cognitive Theory Construct	Linked Behaviour Change Techniques (Theory and Techniques Tool)	Operationalisation in VISUALISE
**Self-Efficacy**		
*Mastery experience*	• Goal setting (behaviour) • Goal setting (outcome) • Feedback on behaviour • Behavioural practice/rehearsal • Graded tasks	• Weekly achievable targets set collaboratively within the group. • Goals broken into small, manageable steps from Session 3 onwards. • Sessions begin with reviewing ‘two positives and one challenge’ from the previous week. • Workbook diary used for ongoing progress documentation. • Between-session tasks (e.g. trial an adaptation, reflect on strengths) provide low-stakes mastery opportunities.
*Vicarious experience*	• Demonstration of behaviour • Social comparison • Credible source	• Peer leaders with lived experience of SRVI share experiences of adapting to stroke-related visual impairment, modelling successful adaptation and strengthening participants' belief in their own capacity to manage similar challenges • Peer supporters share personal recovery milestones, enabling participants to identify with comparable others. *Note: Vicarious experience as a source of self-efficacy shares mechanisms with observational learning (see below); both processes operate through peer modelling but serve distinct functions, with one primarily strengthening confidence and the other facilitating skill acquisition.*
*Improving physical & emotional states*	• Reduce negative emotions • Social support (unspecified)	• 30-minute informal pre-session social time to ease anxiety and build comfort. • Quiet room available throughout sessions. • Pre-programme readiness assessment identifies additional support, accessibility, or tailoring requirements • 1:1 support from peer leaders and a health professional is available before/after sessions.
*Verbal persuasion*	• Social reward • Feedback on behaviour • Credible source	• Praise and encouragement embedded throughout sessions. • Facilitators model goal-setting by sharing their own weekly targets. • Workbook reflection pages reinforce positive feedback loops.
**Observational Learning**		
*Vicarious learning (acquisition of new behaviours and strategies)*	• Demonstration of behaviour • Social comparison • Credible source	• Peer-led delivery enables direct observation of adaptive coping strategies and practical skills (e.g. environmental navigation, aid use, service access). • Weekly target-sharing provides repeated opportunities to observe how others approach and solve similar challenges. • Balanced peer vignettes in the workbook that acknowledge both ongoing challenges and positive experiences of adapting to stroke-related visual impairment
**Outcome Expectations**		
*Social outcome expectations*	• Credible source • Goal setting (behaviour) • Social support (unspecified) • Social reward	• Weekly target review creates a predictable routine of social accountability. • Peer supporters share experiences of adapting to stroke-related visual impairment. • Group ground rules (Session 1) reinforce mutual encouragement. • Weekly praise and recognition following goal review reinforces the social value of participation and progress. • Final celebration session (Session 9) acknowledges individual and group achievements.
*Self-evaluative outcome expectations*	• Self-monitoring of outcomes • Goal setting (outcome) • Framing/reframing	• Workbook encourages first-person documentation of goals and progress. • ‘Two positives and one challenge’ promotes strengths-focused reflection. • Celebration session (Session 9) consolidates perceived personal progress.
**Behavioural Capability**		
*Increasing knowledge & skills*	• Instruction on how to perform a behaviour • Information about health consequences • Information about consequences (emotional, social, environmental) • Demonstration of behaviour • Behavioural practice/rehearsal	• Session 1 provides education on SRVI via presentations, discussion, stories, and role-play. • Session 6 offers hands-on practice with assistive technology. • Multiple delivery modes support cognitive and perceptual diversity. • Workbook provided in accessible formats as an ongoing reference.
**Self-Regulation**		
*Goal setting*	• Goal setting (behaviour) • Goal setting (outcome) • Action planning • Problem solving	• Session 3 introduces structured goal-setting written in the first-person. • Long-term goals translated into weekly targets. • Weekly target-sharing enhances accountability. • Session 4 links goals to specific community services and supports.
*Self-monitoring*	• Self-monitoring of behaviour • Self-monitoring of outcomes	• Workbook diary used to record progress weekly. • Reflection sessions (Sessions 5 and 8) support consolidation of progress. • ‘Two positives and one challenge’ embeds weekly self-review.
*Feedback from others*	• Feedback on behaviour • Social support (unspecified) • Social reward	• Weekly feedback and encouragement from peers and facilitators follow target-sharing activities • Pre-session social time supports informal feedback. • Optional 1:1 support available before and after sessions.
*Enlisting social support*	• Social support (practical) • Social support (emotional) • Social support (unspecified)	• Optional booster sessions (in-person/online/phone/email) sustain peer contact. • Participants may join wider peer networks (e.g. WhatsApp/Facebook). • Cross-cohort networking encourages longer-term connections. • Optional caregiver involvement supported.

#### Stage 4: Programme organisation

VISUALISE consists of nine weekly, peer-led sessions supported by a tailored workbook. Sessions progress from foundational knowledge and group cohesion to goal-setting, practical problem-solving, and planning for long-term adaptation. Table 5 presents an overview of the VISUALISE intervention using the TIDieR template, while the complete TIDieR specification, including supporting stakeholder quotations and implementation detail, is provided in Supplementary Table S3.

**Table 5.** VISUALISE intervention overview and proposed session outline using the TIDieR checklist (Hoffmann et al., 2014).

TIDieR Component/Session	Description	Rationale	Supporting Evidence/Quotations
Brief Name	VISUALISE, a provisional intervention name that may be adapted by each group during Session 1 to reflect their shared goals and identity	Gives participants ownership; ensures the name is meaningful to each specific group.	*“I wonder if there's just that opportunity within this first session to say, you know, in the group, what do you want to call this?”* (Academic)
Why	Developed to address unmet SRVI needs identified in qualitative research; informed by Social Cognitive Theory	Aligns programme with behaviour‑change theory and participant‑identified needs.	See Logic Model (Table 3) and SCT-BCT mapping (Table 4).
Materials	Workbook with session summaries, support services, resources, peer stories, and goal/reflection diary. Available in accessible formats.	Intended to support learning, reflection, strengthening confidence, and long-term reference	*“A workbook and the diary I always say is really important. When I was out of hospital…If it wasn't written down, I probably wouldn't have done it. But also, then looking back, where I'd written, what I've done…this allowed you to reflect”* (Lived Experience)
Pre-intervention assessment	A peer leader and health professional discuss goals, accessibility requirements, support needs, caregiver involvement preferences, and readiness to engage in group-based self-management activities.	Intended to support readiness for participation, identify accessibility needs, and tailor programme delivery.	*“Well, what will happen is that they will come to the group, it'd be a little bit too overwhelming for them. There'll be some emotional issues, and then they won't come back. So, I would say like a dual decision. The clinician and someone who's got some lived experience.”*(Lived experience)
Who Provided	Primarily led by peer leader with support from peer supporters (individuals with lived experience of SRVI) and a health professional (occupational therapist, stroke nurse, or low vision rehabilitation officer).	Peer leaders and supporters are intended to provide relatable examples of adaptation and self-management; ownership by peers rather than health professionals.	“when I wanted to meet people that had a stroke, I just wanted to meet somebody who had made a sort of reasonable recovery…we've got to really be careful about the balance” (Lived experience)
How	Group-based intervention with 6–9 participants. Peer leaders, supporters, and health professional available for 1:1 support before, during, and after each session for goals, weekly targets, and other needs. Peer supporters are encouraged to sit alongside different participants at each sessiondifferent participants each session to build relationships across the group.	Group format is intended to facilitate peer connection; entering a group with strangers can be daunting, peer supporter presence could help participants feel more at ease within the group.	“for people with stroke, sitting in a large group can be difficult for people…how they broke it down was really, really good as well, at the beginning of each session, the peer support who were there, sit beside different people each week. So, you maybe have somebody on your right and somebody on your left”(Lived experience)
Where	Community setting with good transport links (hospital, community centre, school, college, or university). A suitable venue should have car parking, an accessible toilet, a first aider, three rooms (a main session room, a caregiver space, and a quiet room for overwhelmed participants), a horseshoe seating layout with rotating seats, and an option for remote participation via a pre‑practised Zoom connection (though face‑to‑face is preferred).	Different visual impairments require careful room layout; rotating seating promotes relationship-building; remote option supports those with transport barriers or living rurally.	*“the layout of the room…when people got a visual impairment, you're going to have…lots of different people with different sight conditions. So, you will have to have a good idea about…what everyone's condition is so that you can lay the room out so everyone can actually see each other…It's really crucial”*(Lived experience)
When & How Much	Nine sessions, 2 hours each (including 10–15-minute comfort break), weekly for nine consecutive weeks. Participants welcomed 30 minutes before start time for late arrivals and protected social interaction. Session structure: recap previous session at beginning; conclusion at end summarising what was covered and learned.	Two hours allows settling into group and covering content; one hour too short and not worth travel time; two weeks between sessions too far apart for retention. A one-week interval between sessions was considered optimal for translating the content into daily practice.	*“Two weeks is probably too far apart. And at the beginning of the session, there should be a recap on the previous session…and then at the end the session…A conclusion…what we should have covered and maybe what we should have learned”—Lived experience* *“you might find the thing (intervention session) is only getting started and then it's got to finish (if only one hour)”*(Lived experience)
Tailoring	a Intervention delivery is guided by individual participant needs, preferences, and priorities. 1:1 support provided for other SRVI-related needs and concerns before and after each session. Person-centred approach to goal documentation based on participant preference rather than rigid quantitative scales.	Individual needs vary widely; flexibility ensures the intervention is responsive to participants. Person-centred goal documentation respects individual agency.	*“measuring goals…the variables that contribute to that can be immense…quite often it's really important that it's the person that decides whether they've achieved it or not”*(Clinical Psychologist)
Intervention sessions			
Session 1: Welcome & Education	• Introductions and ground rules • Education on SRVI (nature, cause, impact) and self-management • Delivered via workbook, peer discussion, stories, and role-play Homework: reflect on personal strengths and skills	Addresses fundamental lack of understanding of SRVI; prepares participants to identify challenges and seek help; sets the tone for group norms and peer-led delivery.	*“I think that asking people suddenly to reflect on their strengths and skills…will be really difficult for people just on the spot, especially in front of other people. So, kind of maybe setting people a kind of a wee task to reflect on over the week”* (Clinical Psychologist)
Session 2: Strengths & Skills	• Identifying retained strengths and skills post-stroke • Small group work with peer supporters to share skills • Emphasis: 'life has changed, but it is not over' • Feedback to wider group Homework: reflect on what is important and enjoyable	Loss of self-confidence is common after stroke; identifying skills and strengths is intended to support confidence and engagement in subsequent goal-setting activities	“identifying what they can do, and what they want to do is really helpful in terms of writing it down or just recording it in some way. And then they can…be able to go back over and reflect on their progress”(Occupational Therapist)
Session 3: Goal Setting	• Goals set according to what is important to each participant • Goals documented in workbook in first person, in participant's own words • Goals broken into smaller steps with facilitator support • Set weekly target to share with group; facilitators/peer leaders share their own targets From this point onward, each session begins with a review of two positives and one challenge.	Equipped with knowledge of SRVI and personal strengths, participants are better positioned to set goals; the routine of weekly commitment promotes reflection on progress; emphasis on positives rather than negatives.	*“it's really just to reinforce to people the positive, rather than the negative all the time… What went well? I went out and I cut some of the grass…I didn't manage to cut all the grass, but I managed to cut some of it”* (Lived Experience)
Session 4: Accessing Support	• Identifying and accessing available support and services • Link community support and services to individual goals • All services organised by local authority, NHS board, and third-sector provider	No need to duplicate what is already available; efficient to signpost to existing services that can support as needs and goals change over time.	“I find trying to figure out the low vision services for stroke survivors in Aberdeen is different from Glasgow is different from Clydebank…it's an overlap of NHS areas and council borders…And then charities maybe have their own borders…It's hard to understand”(Academic)
Session 5: Reflection I	• First structured reflection session • Share two positives and one challenge • Peers and facilitators support each other to discuss overcoming challenges • Protected time to document progress in workbook and review previous entries 1:1 support available before/after session	Reflection was viewed by stakeholders as an important mechanism for recognising progress and identifying ongoing challenges (and was considered by stakeholders to potentially be an emotionally demanding session requiring optional 1:1 support)	*“when you start writing down, everything that has gone on, you get a better balance of the positive things and the things that have improved…If you don't write it down in the balanced way you just focus on the challenges and the issues and may get into a bit of a loop”*(Lived experience)
Session 6: Technology & Devices	• Facilitated by technology expert supported by intervention facilitators • Opportunity to trial different aids and receive signposting to access points and funding • Participants bring own devices (phones, laptops) for adjustments (font size, brightness, colour)	Technology and assistive devices recognised as wide-ranging and important topic requiring a dedicated session.	“people could bring along their own iPhones or their own laptops? And if there's an expert there…Well, actually, I think that changing, we could try changing the colour of the screen…rather than just seeing lots of other stuff that they need to buy”(Academic)
Session 7: Long-Term Support	• How can I support myself long-term? • Re-emphasise skills and strengths from Session 2 and how they can be utilised beyond the programme • Peer leaders outline their own recovery, service navigation, and how needs/goals changed throughout	Participants may find it difficult to support themselves long-term and to access support for changing needs; prepares for intervention ending.	“this group ends in a couple of weeks time, I can now see some other places to go…rather than the course ending and going off a cliff, it just sort of starts people thinking about their next steps” (Lived experience)
Session 8: Reflection II + Driving (Optional)	• Second reflection session (first hour), following Session 5 format • Optional driving session (second hour): standardised, up-to-date criteria for returning to driving • 1:1 support and peer discussion available	Final reflection useful for reviewing all learning and addressing outstanding issues; driving is a standalone optional session because it would disturb flow for those uninterested but is considered too important to omit entirely.	“with driving, it's such a controversial area that causes so much upset and emotional distress…it needs to be someone that really knows the ins and outs…driving isn't a grey area, it's a black and white area…you need to know that, and you need to look at alternatives”(Occupational Therapist)
Session 9: Closing/Celebration	• Reflect on and celebrate progress as a peer group • Address outstanding questions • Session extended by 30–60 minutes to accommodate emotional processing of programme ending • Prepare participants to maintain peer connections and self-management beyond the programme	Supports participants to reflect on progress and plan to use identified skills, strengths, and available support long-term; acknowledges the emotional impact of the programme ending.	“looking back, where I'd written (in diary), what I've done, and where I've managed to get, I think just measuring it, looking back and seeing what progress you've made is so powerful”(Lived experience)
Booster Sessions (Optional)	• Format decided by each group: face-to-face, Zoom, phone, or email • Option to share contact details and join wider peer network (WhatsApp/Facebook) • Enables connection with participants from other cohorts	Importance of accessing long-term support and maintaining peer relationships; enables peers to remind each other of content, reducing reliance on professionals.	“I think a lot of people struggle, they organise to go to this intervention and then it just stops. And I think, people feel really quite isolated and alone. But even just having the option of some kind of boosting session or being able to make contact would probably be very reassuring” (Low vision rehabilitation ifficer)

#### Key implementation considerations

To support accessibility, engagement, and psychological safety, VISUALISE incorporates several core design features.

Stakeholders did not recommend a fixed post-stroke timeframe for participation. Instead, VISUALISE was designed as a flexible intervention that could be accessed following identification of SRVI when individuals felt ready to engage with group-based self-management support. A joint pre-programme assessment by a peer leader and health professional is intended to identify individual goals, accessibility requirements, support needs, caregiver involvement preferences, and readiness to engage in group discussion, peer interaction, reflection, and goal-setting activities. Stakeholders viewed this process as a means of supporting participant engagement, tailoring intervention delivery, and identifying any additional preparation or support requirements rather than determining eligibility for participation.

Each session is preceded by 30 minutes of informal peer interaction intended to facilitate trust-building and normalise shared experience. Peer‑led delivery, supported by health professionals, was selected because stakeholders believed it may enhance modelling, relatability, and self-efficacy. Facilitator training is role‑specific: all facilitators would require in‑depth knowledge of SRVI and local services; health professionals require experience in collaborative goal‑setting and group facilitation and peer leaders receive preparation in sharing balanced, realistic narratives to minimise unhelpful comparison.

Venues should be selected with consideration for both physical accessibility and psychological comfort. Desirable features include a main room arranged to support visibility and interaction, access to a quiet space for breaks or individual support, and, where appropriate, provision for separate caregiver discussions. Consideration should also be given to accessible parking, suitable toilet facilities, and first-aid arrangements. Face-to-face delivery may be preferable for fostering engagement, although remote participation options can help reduce barriers for individuals who experience transport or mobility challenges.

An accessible workbook is central to the programme. It includes session summaries, structured goal‑setting tools, weekly reflection pages, local service information, and peer stories aligned with Social Cognitive Theory processes. Formats include standard print, large print, aphasia‑friendly, audio, and digital versions, with additional guidance on transport schemes and optional booster sessions to support longer‑term adjustment.

Accessibility considerations extended beyond workbook format alone. Stakeholders proposed multiple methods of information delivery, including verbal discussion, peer modelling, practical activities, repetition of key concepts, workbook prompts, structured reflection, and one-to-one facilitator support (see Table 4).

Caregiver involvement should be tailored according to participant preference and support requirements. These adaptations were intended to support participation among individuals who may also experience stroke-related cognitive, attentional, executive function, or fatigue-related challenges.Caregiver involvement is flexible and based on participant preference. Caregivers may attend information‑focused sessions, but separate spaces are available to support privacy. The approach aimed to balance inclusivity with the need for participants to feel comfortable expressing concerns that may relate to caregiving relationships.

“*Some people might feel actually that it's more difficult to speak about how I feel about my carer, with carers in the room*.”—Clinical Psychologist

Throughout this manuscript, we primarily use ‘individuals living with stroke-related visual impairment’ or ‘participants’ rather than ‘stroke survivor’. This reflects feedback from contributors who felt that the term ‘stroke survivor’ may serve as an unwanted reminder of the stroke event and does not align with all individuals' identities.However, terminology preferences vary widely among individuals, and the intervention invites each group to determine its preferred terminology during Session 1.

#### Potential risks and mitigation

Stakeholders identified several potential risks and corresponding mitigation strategies. Driving was recognised as a particularly sensitive topic, so the driving session is optional and supported by access to quiet rooms and 1:1 discussions. Emotional responses associated with programme ending were also anticipated; Session 9 therefore includes extended reflection and preparation for next steps, with optional booster sessions and peer‑network opportunities to maintain continuity.

Peer support, while central to VISUALISE, carries the risk of unhelpful comparison. To address this, peer leaders are trained to share balanced, realistic narratives that acknowledge ongoing challenges as well as progress. Stakeholders highlighted the importance of balancing these considerations to support confidence and engagement, while recognising that appropriate approaches may differ depending on group composition and peer leader style..

## Discussion

This study presents VISUALISE, a theory‑driven, co‑designed self‑management intervention specifically tailored to the complex psychological, functional and practical challenges associated with stroke‑related visual impairment. To our knowledge, this is the first published intervention development study specifically designed to address stroke‑related visual impairment through an integrated self‑management approach informed by theory, evidence, and lived experience. This intervention responds to well‑documented gaps in care for stroke-related visual impairment, including limited post‑discharge support, inconsistent access to visual‑rehabilitation services, and substantial unmet informational and emotional needs (Rowe et al., 2013).

The rationale for VISUALISE arises from limitations in existing approaches to stroke and vision‑related self‑management. Stroke self-management programmes typically emphasise broad processes including education, collaborative goal-setting, problem-solving, coping and action planning, but these approaches often lack individual tailoring and clear theoretical specification, reducing consistency and reproducibility (Fryer et al., 2016). Vision self-management interventions, by contrast, focus on functional adaptation, including scanning strategies, optical and assistive aids, environmental modification and practical skills training, yet despite their relevance to everyday functioning, show little to no effect on health or vision-related quality of life (Malkin et al., 2025). These gaps highlight the need for an integrated approach that addresses both functional adaptation and the psychological processes underpinning long‑term adjustment.

VISUALISE makes two key contributions. First, the structured application of Intervention Mapping enabled explicit alignment of intervention components with Social Cognitive Theory (SCT). Mapping SCT constructs to Behaviour Change Techniques provides transparency about hypothesised mechanisms of action, an area frequently criticised within self‑management research, and responds to calls for stronger theoretical clarity and improved reporting across stroke (Lau et al., 2022; Lynch et al., 2025) and vision (Malkin et al., 2025) self-management interventions. The VISUALISE TIDieR‑based specification is intended to support replicability and future fidelity assessment.

Second, meaningful, sustained co‑design shaped the structure, accessibility, and delivery of VISUALISE. People with lived experience contributed to decisions regarding emotional safety, session pacing, and hybrid delivery, resulting in key features such as a pre‑session social period, a readiness assessment, balanced peer narratives, and an accessible multi‑format workbook. These elements aim to address the emotional challenges, anxiety, and risk of unhelpful social comparison reported by those with lived experience of stroke-related visual impairment, while strengthening self‑efficacy and social connection. Input from individuals 5–20 years post‑stroke aimed to ensure that the programme reflects both early adjustment needs and longer-term experiences of living with stroke-related visual impairment. This differs from findings reported in the stroke co-design literature, which suggest that co-design activities are frequently focused on early stages of intervention development, while the degree of sustained and iterative stakeholder involvement throughout the research process varies substantially (Singh et al., 2024).

Although VISUALISE was developed within a UK context, primarily from a Scotland perspective, many of its core components are not specific to a single healthcare system. Mechanisms such as peer support, goal-setting, self-efficacy enhancement, behavioural practice, and self-management support are recognised as core elements of self-management interventions across a range of long-term conditions and rehabilitation contexts (Pearce et al., 2016). However, implementation would require contextual adaptation. Consistent with implementation and complex intervention guidance, successful transfer of interventions across settings requires consideration of local service structures, referral pathways, workforce models, funding arrangements, and sociocultural context (Movsisyan et al., 2021; Skivington et al., 2021). Consequently, VISUALISE may be viewed as a transferable intervention framework, comprising core functions that may be retained across settings and adaptable components that can be modified to reflect local service pathways, resources, and implementation contexts.

Several limitations should be acknowledged. Online co‑design during COVID‑19 may have excluded people with limited digital access or confidence, reducing diversity of input. The predominantly Scottish stakeholder group may limit transferability, and context‑specific components (e.g. service directories) will require adaptation. Two stakeholders, a stroke rehabilitation nurse and a caregiver, contributed via email rather than participating in focus group discussions. While this enabled inclusion of perspectives that might otherwise have been missed, email contributions did not allow for interaction with other participants or the co-construction of ideas. Consequently, these responses reflected individual perspectives and may have differed from insights generated through group discussion.

An imbalance between professional and lived‑experience contributors, and pre‑existing relationships between advisors and researchers, may also have shaped perspectives. Lived-experience contributors had previously participated on the wider advisory group supporting the research programme and therefore had established relationships with the research team. While this continuity facilitated sustained engagement and informed contributions to intervention development, it may also have introduced relationship bias, whereby familiarity with researchers' perspectives and priorities influenced discussions. Future intervention-development studies may benefit from combining ongoing contributors with newly recruited stakeholders to balance continuity with independent perspectives. Additionally, some perspectives, particularly those of individuals with more complex visual perceptual presentations or those in earlier stages of recovery, may be underrepresented. Future refinement and evaluation should seek input from a broader range of individuals living with stroke-related visual impairment.

Although VISUALISE incorporates several adaptations intended to support participation, including multimodal information delivery, facilitator support, structured reflection, one-to-one support, and optional caregiver involvement, individuals experiencing more severe cognitive impairment following stroke were not represented within the co-design process. Consequently, the accessibility, acceptability, and support requirements of VISUALISE for these groups remain uncertain and should be explored during future feasibility, implementation, and evaluation studies. Finally, although mapping components to Social Cognitive Theory clarified proposed mechanisms, real‑world delivery may produce overlapping effects that make specific drivers of change difficult to isolate. Future feasibility, process and effectiveness evaluation will be essential. Intervention Mapping is resource‑intensive, and the depth of specification achieved may exceed what some services can easily implement without support. Similarly, SRVI services vary substantially across geographic regions, and the intervention’s signposting content will require local adaptation.

The study did not undertake implementation or evaluation planning (Intervention Mapping Stages 5–6), meaning that the feasibility, acceptability, and effectiveness of VISUALISE in practice remain to be established. Consistent with the aims of Intervention Mapping, this study focused on intervention development rather than evaluation. Consequently, the proposed mechanisms through which VISUALISE may influence outcomes are theoretically informed and stakeholder proposed hypotheses that require testing through future feasibility, process, and effectiveness studies. Future work should progress these stages by developing a peer‑leader training package, refining intervention materials, and conducting feasibility testing. Implementation research will also be needed to determine scalability, contextual adaptation, and cost‑effectiveness across diverse stroke and vision‑rehabilitation pathways.

## Conclusion

In summary, VISUALISE is a theoretically grounded, co‑designed intervention that is designed to address longstanding gaps in support for people living with stroke‑related visual impairment. The proposed intervnetion's transparent theoretical foundation, detailed specification, and integration of lived‑experience insight provide a basis for feasibility testing and represent a methodological contribution to the development of self‑management interventions.

## Supplementary Material

Supplementary Material Supplementary_material_FINAL_-_24-10-16___1_.docx

## Data Availability

The datasets used and/or analysed during the current study are available from the corresponding author on reasonable request.
