# Supplementary material for: The development of VISUALISE: a co-designed, theory-based self-management intervention for stroke-related visual impairment
Source: Health Psychol Behav Med. 2026 Nov 7;14(1):2753947. doi: 10.1080/21642850.2026.2753947 (PMC13757788; doi:10.1080/21642850.2026.2753947)
Supplement: Supplementary file 1 [file RHPB_A_2753947_SM9995.docx]

## Supplementary Material

**Table S1:** Integrated Logic Model of the Problem and Proposed Intervention Solutions

**Table S2:** Application of Social Cognitive Theory to VISUALISE

**Table S3:** Full TIDier Description of VISUALISE

## Table S1: Integrated Logic Model of the Problem and Proposed Intervention Solutions

| **Priority Domain** | **Key Personal Determinants** | **Key Environmental Determinants** | **Proposed Intervention Solutions (VISUALISE component)** |
| --- | --- | --- | --- |
| **Information Provision & Education** | - Limited understanding of SRVI (nature, causes, functional consequences) - Difficulty articulating SRVI-related challenges to others - Unawareness of available support options and how to access them - Low self-efficacy to seek help or request reasonable accommodations | - Generic, inaccessible, or passively delivered information - Poor timing of information provision (e.g. at acute hospital discharge, before difficulties are fully apparent) - Limited awareness of SRVI among clinicians and the public - Gap in care between hospital discharge and community service access - Regional inequity in NHS and third-sector provision - Key resources not routinely signposted (e.g. free travel pass, DVLA processes, Citizens Advice) | - Face-to-face education with SRVI-informed professionals (Session 1) - Accessible, repeated information with time for questions (Session 1; accessible-format workbook) - Role-play or peer support to build confidence (Sessions 1–2) - Highlight key resources (e.g. travel pass, DVLA, Citizens Advice) (Session 4; workbook) - Provide postcode-specific service information (Session 4; workbook services listed by local authority) |
| **Practical Support** | - Low self-efficacy to trial or adopt assistive aids and adaptive strategies - Self-taught strategies reduce confidence - Fear of stigma or perceived vulnerability linked to visible aids - Identity disruption related to aid use - Concerns about others’ reactions when using aids in public - Assumptions about IT/digital literacy acting as barriers - Stroke-related co-impairments limiting device use - Pre-stroke technology experience shaping adjustment | - Insufficient therapy time to practise with aids before/after discharge - Lack of demonstration or supported trial opportunities - Financial and resource barriers to acquiring aids and devices | - Involve caregivers to boost confidence (optional caregiver involvement) - Hands-on trial of aids and adjustment of participants’ own devices (Session 6) - Signpost aids and funding sources (Session 6) - Refer to support services (e.g. Visibility Scotland) (Sessions 4 and 6) |
| **Peer & Emotional Support** | - Strong desire for peer connection with others who share lived experience - Pre-existing reluctance or anxiety about peer engagement due to fear of unfavourable social comparison - Misunderstanding of SRVI progression and long-term prognosis - Emotional distress associated with abrupt driving licence loss | - Lack of clarity around DVLA notification and assessment processes, causing prolonged uncertainty - Time-limited peer groups with no sustained contact or follow-up - Insufficient professional reassurance delaying psychological adjustment | - Protected time for peer support (30-minute pre-session social time; optional booster sessions) - Balanced, realistic peer narratives to minimise unhelpful comparison (peer leader training; workbook vignettes) - Clear DVLA information followed by emotional support (Session 8, optional driving hour) - 1:1 time with professionals for concerns (1:1 support before/after each session) |
| **Confidence (cross-cutting domain)** | - Low self-efficacy to seek help, self-advocate, or use assistive aids - Reduced sense of identity and self-worth after stroke - Difficulty recognising and valuing retained abilities and personal strengths - Limited opportunity or encouragement to focus on strengths rather than losses | - Limited understanding of SRVI within social networks and family - Absence of structured, ongoing opportunities to reflect on and celebrate progress | - Focus on retained abilities (e.g. journaling) (Session 2; workbook diary) - Improve understanding of stroke-related visual impairment and personal challenges (Session 1) - Use reflection to track progress (Sessions 5, 8 and 9; weekly “two positives and one challenge”) |

## Table S2: Application of Social Cognitive Theory to VISUALISE

| **Social Cognitive Theory Construct** | **Sub-construct** | **Practical Applications** | **Supporting Evidence** |
| --- | --- | --- | --- |
| **Self-efficacy** | Mastery experience | - Set weekly achievable targets with peer feedback - Document weekly progress in the intervention workbook | - *"have those really specific goals, like every week...so like something that they're definitely going to be able to achieve" (Clinical Psychologist)* - *"Because if you can see that written down, it's so much more helpful" (Clinical Psychologist)* |
|  | Improving physical/emotional states | - Allow 30 minutes pre-session for participant interaction and relaxation | - *"We have like a pre-meeting...where half an hour before the group actually formally starts people rock up and have a chat...everyone's chatting with a cup of tea" (Lived Experience)* |
|  | Verbal persuasion | - Praise and encouragement from peers and facilitators - Document and reflect on progress in workbook | - *"looking back, where I'd written, what I've done...I can walk the pillar box now. Whereas before I couldn't walk the front door" (Lived Experience)* - *"if we can get a feedback for someone who is important in our life, that may support any progress" (Eye Clinic Liaison Officer)* |
|  | Social modelling | - Use peer leaders to lead the intervention - Share lived experiences among peers | - *"we don't want it to be...driven by health professionals...but stroke survivors, peers so they really feel that they have ownership" (Academic)* - *"this happened to me...you might think that things are really bad...but things are going to get better" (Lived Experience)* |
| **Observational learning** | Observational learning | • Group sharing of weekly targets  • Use balanced peer vignettes | - *"learning from others is one of them, getting feedback from credible sources...success stories, sharing experiences" (Occupational Therapist)* - *"storytelling aspect...allows people to almost see themselves in those stories...it's more of that peer approach" (Academic)* |
| **Outcome expectations** | Social outcome expectations | • Review weekly achievable targets to create routine and accountability | *"I'm going to commit to do this this week, and I'm going to have to feedback to everybody...They're getting that routine throughout the weeks" (Clinical Psychologist)* |
|  | Self-evaluative outcome expectations | • Encourage first-person documentation of progress in workbook | *"my diary was Monday, I'm going to go for a walk...If it wasn't written down, I probably wouldn't have done it...looking back...this allowed you to reflect" (Lived Experience)* |
| **Behavioural capability** | Increase knowledge | • Provide mixed intervention methods: workbook, presentations, peer discussion, role-play | *"having a mixture of delivery would be really good...peer group discussion...in pairs, and discuss aspects...then feedback" (Clinical Psychologist)*  *"a workbook along with a presentation...allows people to reflect back" (Lived Experience)* |
| **Self-regulation** | Goal setting | • Weekly goal setting alongside longer-term goals  • Identify support services to reach goals | *"it's about person centeredness, and goal setting...It's about just what do I want to do? What do I need to do?" (Occupational Therapist)*  *"there's a lot of those resources out there...making sure that...stroke services, alert service users" (Occupational Therapist)* |
|  | Self-monitoring | • Use the diary section in the workbook to document progress | *"A workbook and the diary I always say is really important...people who I see every two weeks, who have made a massive improvement, but they don't see it" (Lived Experience)* |
|  | Feedback from others | • Weekly praise from peers and facilitators  • Protected time for peer support | *"allowing that time for people to relax enough to be able to find who their people are and be able to build that peer network" (Academic)*  *"the peer discussion is a really helpful one. Because a lot of people do get a lot of information from each other" (Occupational Therapist)* |
|  | Enlistment of social support | • Option to join wider peer network | *"trying to promote that sort of peer support...if people are happy for us to put that out to the group...kind of keep in that contact themselves...using that peer support" (Clinical Psychologist)* |

## Table S3: Full TIDier Description of VISUALISE

| **TIDieR Component / Session** | **Description** | **Rationale** | **Supporting Quotes/ Evidence** |
| --- | --- | --- | --- |
| **Pre-intervention Assessment** | Completed by peer leader and a health professional to gather:  1. Participant’s challenges and goals  2. Accessibility needs  3. Caregiver involvement  4. Readiness to engage | To ensure participants are ready to engage and avoid unfavourable comparisons that could damage confidence; enables facilitators to plan content and tailor materials accordingly | *"Well, what will happen is that they will come to the group, it'd be a little bit too overwhelming for them. There'll be some emotional issues, and then they won't come back. So, I would say like a dual decision. The clinician and someone who's got some lived experience." (Lived experience)* |
| **Brief Name** | *VISUALISE - a generic name that can be changed by each intervention group during the first session to reflect what engagement means to them* | To give participants ownership and ensure the name is meaningful to their specific group | *"I wonder if there's just that opportunity within this first session to say, you know, in the group, what do you want to call this?"* (Academic) |
| **Why** | To address priority unmet needs derived from a previous qualitative study. The intervention is informed by Social Cognitive Theory (SCT), specifically addressing constructs of self-efficacy, observational learning, outcome expectations, behavioural capability and self-regulation | Based on systematic identification of unmet needs and theoretical framework to support self-management behaviour change | |
| **Materials** | Workbook includes:  • Session overview  • Local supports/services  • Key resources  • Peer stories  • Diary for goals and reflection   Features:  • Jargon-free, simple language  • Accessible formats (print, online, audio, aphasia-friendly) | Enables participants to follow session content, document thoughts and goals, support reflection on progress, increase outcome expectations, and boost self-efficacy (SCT constructs); supports sustainability as participants can refer back as needs evolve; no need to "reinvent the wheel" - signpost to existing services | *"there's a lot of those resources out there, but perhaps not everybody knows about them ... it's a shame if people don't know that they're there because they can be helpful"* (Occupational Therapist)  *"A workbook and the diary I always say is a really important. When I was out of hospital ... If it wasn't written down, I probably wouldn't have done it. But also, then looking back, where I'd written, what I've done ... this allowed you to reflect"* (Lived Experience) |
| **Session 1: Welcome & Education** | Introductions, ground rules, education on stroke related visual impairment and self-management. Delivered via workbook, peer discussion, stories, and role-play. Homework: reflect on strengths and skills | Addresses lack of understanding of stroke related visual impairment; prepares participants to identify challenges and seek help | *"I think that asking people suddenly to reflect on their strengths and skills ... will be really difficult for people just on the spot, especially in front of other people. So, kind of maybe setting people a kind of a wee task to reflect on over the week"* (Clinical Psychologist) |
| **Session 2: Strengths & Skills** | Identifying strengths and skills. Participants work in groups with peer supporters to share skills and strengths. Emphasis on "life has changed, but it is not over." Feedback to wider group.  Homework: reflect on what’s important and enjoyable | Loss of self-confidence occurs after stroke; identifying skills and strengths improves confidence ahead of goal-setting session | *"identifying what they can do, and what they want to do is really helpful in terms of writing it down or just recording it in some way. And then they can ... be able to go back over and reflect on their progress"* (Occupational Therapist) |
| **Session 3: Goal setting** | Goal setting according to what's important to each participant. Goals documented in workbook in first person, participant's own words. Break goals into smaller steps with facilitator support. Set weekly target to share with group. Facilitators share their own targets.  From this session onwards: each session begins with sharing two positives and one challenge from previous week | Equipped with knowledge of visual impairment and personal strengths, participants better positioned to set goals; routine of commitment promotes reflection on progress; emphasis on positives rather than negatives | *"it's really just to reinforce to people the positive, rather than the negative all the time. I can't do that ... And it's just to emphasise the positive. What went well? I went out and I cut some of the grass ... I didn't manage to cut all the grass, but I managed to cut some of it"* (Lived Experience)  *"almost like everybody setting a goal at the end of the session ... tiny, so like something that they're definitely going to be able to achieve. But just to start them in that routine of, I'm going to commit to do this this week ... They're getting that routine throughout the weeks"* (Clinical Psychologist) |
| **Session 4: Accessing Support** | Identifying and accessing available support. Link community support and services to individual goals. All services broken down by local authority and third sector in workbook for ongoing reference | No need to duplicate what's already available; efficient to signpost to existing services that can support as needs and goals change over time | *"I find trying to figure out the low vision services for stroke survivors in Aberdeen is different from Glasgow is different from Clydebank ... it's an overlap of NHS areas and council borders ... And then charities maybe have their own borders ... It's hard to understand"* (Academic) |
| **Session 5: Reflection I** | First reflection session. Share two positives and one challenge. Participants and facilitators support each other to discuss overcoming challenges. Protected time to document progress in workbook and reflect on previous entries. One-to-one support available before/after session | Reflection essential for considering progress and recognising outstanding challenges requiring further support; potentially emotive topic requiring additional support | *"when you start writing down, everything that has gone on, you get a better balance of the positive things and the things that have improved ... If you don't write it down in the balanced way you just focus on the challenges and the issues and may get into a bit of a loop"* (Lived Experience) |
| **Session 6: Technology & Devices** | Technology and devices session. Facilitated by technology expert supported by intervention facilitators. Opportunity to try different aids, receive signposting to access points and funding sources. Participants bring own devices (phones, laptops) for adjustments (font size, brightness, colour) | Technology and devices recognised as wide-ranging and important topic requiring full session | *"people could bring along their own iPhones or their own laptops? And if there's an expert there ...Well, actually, I think that changing, we could try changing the colour of the screen ... rather than just seeing lots of other stuff that they need to buy, or could cost money that they bring their own stuff. And that can be tailored to them"* (Academic) |
| **Session 7: Long-Term Support** | How can I support myself long-term? Re-emphasise skills and strengths from Session 2 and how they can be utilised beyond the programme. Peer leaders outline their own recovery, accessing supports/services, and how needs/goals changed throughout | Difficulty supporting themselves long-term and accessing support for changing needs; prepare for intervention ending | *"this group ends in a couple of weeks time, I can now see some other places to go ... rather than the course ending and going off a cliff, it just sort of starts people thinking about their next steps, really, because this group can't last forever"* (Lived Experience) |
| **Session 8: Reflection II + Driving (Optional)** | Second reflection session (first hour) following Session 5 format. Optional driving session (second hour) with standardised up-to-date criteria for returning to driving. One-to-one support and peer discussion available | Final reflection useful for reviewing all learning and addressing outstanding issues; driving standalone session would disturb flow for those uninterested, so made optional during second hour | *"with driving, it's such a controversial area that causes so much upset and emotional distress ... it needs to be someone that really knows the ins and outs ... driving isn't a grey area, it's a black and white area ... you need to know that, and you need to look at alternatives ... allowing people to have some sort of discussion and ask the individual questions as well"* (Occupational Therapist) |
| **Session 9: Closing/Celebration** | Closing/celebration session. Reflect on and celebrate progress as peer group. Address outstanding questions. Provision for session to be 30-60 minutes longer for additional support as participants may be emotional about intervention ending | Support participants to reflect on progress and how to use identified skills, strengths, and available support long-term; acknowledge emotional impact of ending | *"But also then looking back, where I'd written (in diary), what I've done, and where I've managed to get, I think just measuring it, looking back and seeing what progress you've made is so powerful"* (Lived experience)  *"the idea that this is this is only the beginning. So, you don't feel like I've got to the end of the group and I didn't quite get where I wanted to be ... It's like this is this is just the start now you've got the tools to kind of keep going"* (Clinical Psychologist) |
| **Booster Sessions** | Optional booster sessions with format decided by each group (face-to-face, Zoom, phone, email). Option to share contact details and join wider peer network (WhatsApp/Facebook) to maintain relationships and make new connections with other intervention participants from different cohorts | Importance of accessing long-term support and maintaining peer relationships; enables peers to remind each other of content, reducing reliance on professionals | *“I think a lot of people struggle, they organise to go to this intervention and then it just stops. And I think, people feel really quite isolated and alone. But even just having the option of some kind of boosting session or being able to make contact would probably be very reassuring"* (Low Vision Rehabilitation Officer)  *"what you would like is for people to be able to kind of keep in that contact themselves. And using that peer support and kind of reminding each other of what was covered in the group sessions, rather than relying on coming back to the professional"* (Clinical Psychologist) |
| **Who Provided** | Primarily led by peer leader with support from peer supporters (individuals with lived experience of SRVI) and health professional (occupational therapist, stroke nurse, or low vision rehabilitation officer)  Training requirements:  • All facilitators: in-depth knowledge of stroke, SRVI, relevant support/services  Health professional: previous experience with goal setting and group facilitation  Peer leaders/supporters must provide 'balanced' approach - not too positive (damaging to confidence of those with less progress) or too negative  Peer leaders/supporters: balanced approach (not overly positive or negative) | Peer leader and supporters serve as 'models' for participants and provide key source of peer support; ownership by peers rather than health professionals | *“we don't want it to be driven by health professionals or led by health professionals, but stroke survivors and peers so they really feel that they have ownership over this programme for them"* (Academic)  *"when I wanted to meet people that had a stroke, I just wanted to meet somebody who had made a sort of reasonable recovery ... If you introduce people to people who are quite badly disabled ... or somebody that's almost seems completely normal ... it'll affect their confidence because they'll think well, how can I ever aspire to that ... we've got to really be careful about the balance"* (Lived Experience) |
| **How** | Group-based intervention with 6-9 participants. Peer leaders, supporters, and health professional available for one-to-one support before, during, and after intervention sessions for goals, weekly targets, and other needs. Peer supporters available to ease transition into group and support forming of peer relationships | Group format supports peer connection; entering group with strangers can be daunting; peer supporter presence eases transition and supports comfort | *"for people with stroke, sitting in a large group can be difficult for people ... how they broke it down was really, really good as well, at the beginning of each session, the peer support who were there, sit beside different people each week. So, you maybe have somebody on your right and somebody on your left"* (Lived Experience) |
| **Where** | Community setting with good transport links (hospital, community centre, school, college, or university). Schools/colleges/universities optimal (equipment available: tables, chairs, projectors; good transport links).  Requirements:  • Car parking  • Disabled toilet  • First aider  • Three separate rooms: (1) intervention session room, (2) caregiver meeting space, (3) quiet room for participants if overwhelmed/upset  Room layout: 'horseshoe' format optimal (everyone can see everyone vs. rows). Encourage rotating seats/sitting with different people each session to build relationships across all participants  Remote option: Provision to join via Zoom with practice session beforehand, though face-to-face preferred. Signposting to transport services (voluntary transport Scotland, 'fares for free') in workbook. Peer supporters available to assist from stations to intervention venue | Different visual impairments require careful room layout; rotating seating promotes relationship-building across all participants; remote option supports those who may not have easy transport access/ live rurally | *"when I run these [intervention/ peer support sessions] before, we try to rotate the seating every two or three weeks, because people end up sitting with the same two or three people ... If you mix it up a little bit ... people meet other people with different knowledge and experience ... there's lots of experience in those rooms ... you have to sort of mix them all up to get the best effect"* (Lived Experience)  *"the layout of the room ... when people got a visual impairment, you're going to have ... lots of different people with different sight conditions. So, you will have to have a good idea about ... what everyone's condition is so that you can lay the room out so everyone can actually see each other ... It's really crucial"* (Lived Experience) |
| **When and How Much** | Nine intervention sessions, 2 hours each (including 10–15-minute comfort break for peer socialisation), occurring weekly for nine consecutive weeks.  Participants welcomed 30 minutes before start time for late arrivals and protected social interaction time.  Session structure: Recap previous session at beginning, conclusion at end summarising what covered and learned | Two hours allows settling into group and covering content; one hour may be too short and not worth travel time; two weeks between sessions too far apart for retention. One week optimal for considering content, applying to life, and retaining for next session. Account for late arrivals (public transport issues etc.,) | *"Two weeks is probably too far apart. And at the beginning of the session, there should be a recap on the previous session ... and then at the end the session ... what we should have covered and maybe what we should, should have learned"* (Lived Experience)  *"you might find the thing [intervention session] is only getting started and then it's got to finish"* (Lived Experience)  *“We have like a pre-meeting ... where half an hour before the group actually formally starts people rock up and have a chat"* (Lived Experience) |
| **Tailoring** | Although developed around specific unmet needs, intervention facilitators led by individual participant needs and wishes. Provide one-to-one support for other SRVI-related needs and concerns as required before and after each intervention session.  Person-centered approach to goal documentation based on participant preference (rather than rigid quantitative scales) | Individual needs vary; flexibility ensures intervention responsive to participants | *"measuring goals ... the variables that contribute to that can be immense ... quite often it's really important that it's the person that decides whether they've achieved it or not"* (Clinical Psychologist)  *"I think maybe being person centred is a good way to go forward as in that people deciding themselves is a key function"* (Occupational Therapist) |
| **Modifications and How well (planned/ actual)** | Not applicable as the intervention has not yet been delivered; fidelity to be assessed in feasibility testing. | | |
| **Caregiver Involvement** | Each participant decides individually whether a caregiver is present during sessions.  Benefits of presence:  • Support documenting goals (especially with other stroke impairments)  • Helpful for information-heavy sessions (1, 6) to translate learning into daily life  Drawbacks:  • Participants may not feel able to be as open  • Risk of caregivers taking over/speaking on behalf  If not participating, a separate room is available for informal caregiver peer support while intervention is ongoing | Balancing benefits of caregiver support with allowing participants space to speak openly about their experiences | *"they prefer to have someone with them ... other people might feel actually that it's more difficult to speak about how I feel about my carer or with carers in the room ... that first session ... sounds like that'd be a really useful session for like, caregivers to attend. And maybe the ones about the technology and maybe the more like information heavy"* (Clinical Psychologist) |
| **Additional Factors: Terminology** | 1. Avoid term 'stroke survivor' - constant reminder of stroke incident, labels individuals as 'disabled'  2. Goal-setting terminology (alternatives: values, aims, needs, wishes, expectations, targets). Allow the group to decide terminology for the duration of the intervention | Individuals have different perceptions of terms; individual preference varies widely; optimal approach is group decision | *"I've always struggled with the phrase stroke survivor ... it's a constant reminder that you have survived it ... you don't say heart attack survivor, do you?" (Lived Experience)*  *"people have different perceptions of what those terms mean. And I think that it's worth having that conversation about what, what goals are ... give them the alternatives, like, needs and wishes, and perhaps allow them to make that decision about what they want to call them"* (Occupational Therapist) |
